# A cluster randomized controlled trial for assessing POC-CCA test based praziquantel treatment for schistosomiasis control in pregnant women and their young children: study protocol of the freeBILy clinical trial in Madagascar

**DOI:** 10.1186/s13063-021-05769-6

**Published:** 2021-11-20

**Authors:** Daniela Fusco, Raphäel Rakotozandrindrainy, Rivo Andry Rakotoarivelo, Mala Rakoto Andrianarivelo, Njary Rakotozandrindrainy, Tahinamandranto Rasamoelina, Dewi Ismajani Puradiredja, Philipp Klein, Karl Stahlberg, Marie Dechenaud, Eva Lorenz, Anna Jaeger, Andrea Kreidenweiss, Pytsje T. Hoekstra, Akim Ayola Adegnika, Elisa Sicuri, Paul L. A. M. Corstjens, Govert J. van Dam, Jürgen May, Norbert Georg Schwarz, on behalf of the freeBILy consortium, G. J. van Dam, P. L. A. M. Corstjens, C. J. de Dood, P. T. Hoekstra, A. S. Amoah, M. I. Keshinro, A. Kreidenweiss, N. G. Schwarz, D. Fusco, P. Klein, A. Jaeger, E. Lorenz, A. A. Adegnika, Y. J. Honkpehedji, J. C. Dejon-Agobe, R. Beh Mba, M. Mbong Ngwese, M. Nzamba Maloum, A. Nguema Moure, B. T. Meulah, R. A. Rakotoarivelo, A. Ralaizandry, M. Radomanana, R. Rakotozandrindrainy, N. Rakotozandrindrainy, Marie Jeannine Solonirina, M. Rakoto Andrianarivelo, T. Rasamoelina, R. Razafindrakoto, E. Sicuri, C. Aerts

**Affiliations:** 1 Department of Infectious Diseases Epidemiology, Bernhard Nocht Institute for Tropical Medicine (BNITM), Bernhard-Nocht-Strasse 74, D-20359 Hamburg, Germany; 2 German Center for Infection Research (DZIF), Hamburg-Borstel-, Lübeck, Riems Germany; 3 Department of Microbiology and Parasitology, University of Antananarivo, 101 Antananarivo, Madagascar; 4 Department of Infectious Diseases, University of Fianarantsoa Andrainjato, 301 Fianarantsoa, Madagascar; 5 Centre d’Infectiologie Charles Mérieux (CICM), University of Antananarivo, PO Box 4299, 101 Antananarivo, Madagascar; 6 UPFR in Parasitology-Mycology of University Hospital Joseph Ravoahangy Andrianavalona Ampefiloha, 101 Antananarivo, Madagascar; 7 Institute of Medical Biostatistics, Epidemiology and Informatics, University Medical Centre of the Johannes Gutenberg University Mainz, Mainz, Germany; 8 Institut für Tropenmedizin, Universität Tübingen, Wilhelmstrasse 27, D-72074 Tübingen, Germany; 9 German Center for Infection Research (DZIF), partner site Tübingen, Wilhelmstrasse 27, D-72074 Tübingen, Germany; 10 Department of Parasitology, Leiden University Medical Center, Albinusdreef 2, 2333 ZA Leiden, the Netherlands; 11 Centre de Recherches Médicales de Lambaréné, 242 Lambarene, BP Gabon; 12 ISGlobal, Hospital Clínic - Universitat de Barcelona, Spain C/ Rosselló, 132, 5th 2nd, 08036 Barcelona, Spain; 13 Department of Cell and Chemical Biology, Leiden University Medical Center, Albinusdreef 2, 2333 ZA Leiden, the Netherlands

**Keywords:** Schistosomiasis, Pregnant women, Children, Mass drug administration, Praziquantel, Point of care-circulating cathodic antigen test, Test-based schistosomiasis treatment, Cluster randomized controlled trial

## Abstract

**Background:**

Mass drug administration (MDA) of praziquantel is one of the main control measures against human schistosomiasis. Although there are claims for including pregnant women, infants and children under the age of 5 years in high-endemic regions in MDA campaigns, they are usually not treated without a diagnosis. Diagnostic tools identifying infections at the primary health care centre (PHCC) level could therefore help to integrate these vulnerable groups into control programmes. freeBILy (fast and reliable easy-to-use-diagnostics for eliminating bilharzia in young children and mothers) is an international consortium focused on implementing and evaluating new schistosomiasis diagnostic strategies. In Madagascar, the study aims to determine the effectiveness of a test-based schistosomiasis treatment (TBST) strategy for pregnant women and their infants and children up until the age of 2 years.

**Methods:**

A two-armed, cluster-randomized, controlled phase III trial including 5200 women and their offspring assesses the impact of TBST on child growth and maternal haemoglobin in areas of medium to high endemicity of *Schistosoma mansoni*. The participants are being tested with the point of care-circulating cathodic antigen (POC-CCA) test, a commercially available urine-based non-invasive rapid diagnostic test for schistosomiasis. In the intervention arm, a POC-CCA-TBST strategy is offered to women during pregnancy and 9 months after delivery, for their infants at 9 months of age. In the control arm, study visit procedures are the same, but without the POC-CCA-TBST procedure. All participants are being offered the POC-CCA-TBST 24 months after delivery. This trial is being integrated into the routine maternal and child primary health care programmes at 40 different PHCC in Madagascar’s highlands. The purpose of the trial is to assess the effectiveness of the POC-CCA-TBST for controlling schistosomiasis in young children and mothers.

**Discussion:**

This trial assesses a strategy to integrate pregnant women and their children under the age of 2 years into schistosomiasis control programmes using rapid diagnostic tests. It includes local capacity building for clinical trials and large-scale intervention research.

**Trial registration:**

Pan-African Clinical Trial Register PACTR201905784271304. Retrospectively registered on 15 May 2019

## Introduction

Human schistosomiasis is a neglected tropical disease (NTD) caused by trematode flatworms [1]. Among the NTDs, schistosomiasis ranks highest in terms of disability-adjusted life years (3.3 million DALYs), with more than 230 million people infected and 600 million being at risk of infection worldwide [2]. It is of particular public health importance in Africa where both intestinal and urinary forms of the disease caused by *Schistosoma mansoni* and *Schistosoma haematobium*, respectively, are prevalent. Schistosomiasis is endemic in 76 countries, and 85% of people infected live in rural areas of sub-Saharan Africa [3]. Madagascar is one of the most affected countries [4]. To reduce morbidity, the WHO recommends annual treatment of school-age children with praziquantel (PZQ) in areas of high endemicity. This is often put into practice via mass drug dministration (MDA) campaigns [5]. MDA strategies, however, have some limitations: usually, MDA focuses on school-age children, thus excluding large groups of the affected population from treatment, such as adults, pregnant women and children under the age of 5 years (U5) [6].

The main public health impact of *Schistosoma* infections is associated with chronic evolutions of the disease. Chronic infections with *S. mansoni* can lead to hepatic inflammation that progresses to advanced hepatic disease and can culminate in cirrhosis or cancer [7]. Chronic infections with *S. hematobium* can lead to ectopic pregnancy, infertility, abortion and cervical lesions and symptoms mimicking cervical cancer (female genital schistosomiasis (FGS)) [8]. All schistosomiasis infections can lead to chronic, not-organ-specific symptoms such as anaemia, fatigue and nausea. In children, schistosomiasis manifests unspecifically but equally detrimental with failure to thrive. In pregnant women, schistosomiasis may lead to anaemia.

The scientific community and WHO recognize the need for a holistic and integrated approach as the most effective way to tackle the disease [9]. Unfortunately, the exclusion of adults, pregnant women, infants and U5 children from MDA leads to increased morbidity, the perpetuation of poverty and a of schistosomiasis transmission [5].

Guidelines for the treatment of schistosomiasis during pregnancy are not yet widely implemented. After more than 30 years of post-market experience with praziquantel, no reports of serious adverse events relevant to human pregnancy have ever been published, but results from randomized controlled trials (RCTs) assessing the efficacy and safety of the drug within this target group are scarce [10]. Recent recommendations of the WHO state that schistosomiasis in pregnant women is harmful and signifies an indication for treatment. Likewise, U5 children should be given PZQ if a *Schistosoma* infection can be confirmed by a diagnostic test [5]. Effective diagnostics available at the primary level of care to detect infection are rarely integrated within the health services of the most affected countries [11]. Since 2008, a point of care (POC) rapid diagnostic test (RDT) based on the detection of the schistosome circulating cathodic antigen (CCA) [10], POC-CCA, became commercially available and is widely evaluated in various endemic settings [12–15]. The introduction of this type of tests at the primary level of care could address the treatment of vulnerable groups, if its use in the context of a dedicated strategy can be shown to be effective.

In order to improve the diagnostic strategies for the management of schistosomiasis in vulnerable groups, the international consortium freeBILy (fast and reliable easy-to-use-diagnostics for eliminating bilharzia in young children and mothers) was initiated [16]. The freeBILy consortium carries out two conceptually different trials, one in Madagascar (Universities of Antananarivo and Fianarantsoa and *Centre d’Infectiologie Charles Mérieux*) and the other one in Gabon (*Centre de Recherches Médicales de Lambaréné*) in collaboration with the Bernhard Nocht Institute for Tropical Medicine (BNITM), the Barcelona Institute for Global Health (ISGlobal), the Eberhard Karls Universität of Tübingen and the Leiden University Medical Center.

The freeBILy trial in rural Madagascar is a phase III cluster RCT (CRCT). This CRCT aims to determine the effectiveness of test-based schistosomiasis treatment (TBST) using POC-CCA for pregnant women and their infants. A cost-effectiveness analysis accompanies the trial and compares costs and health impact associated with TBST with the status quo. The cost-effectiveness will generate knowledge in the context of very few economic evaluations conducted on human schistosomiasis interventions [17].

## Methods

### Study objectives

The overall purpose of freeBILy in Madagascar is to integrate a POC-CCA test-based schistosomiasis treatment (TBST) into routine maternal and child primary health care programmes. The study investigates the effectiveness of the strategy for controlling schistosomiasis in young children and mothers.

Specific objectives of freeBILy in Madagascar are as follows:

1. To assess the impact of TBST on child development by comparing schistosomiasis-associated growth and developmental disadvantages among young children in TBST sites (intervention) to those in non-TBST sites (control)
2. To examine the impact of TBST on maternal health by comparing schistosomiasis-associated anaemia among young mothers in TBST sites to those in non-TBST sites
3. To evaluate the sensitivity and specificity of the urine-based POC-CCA test for the detection of *S. mansoni* infections in routine maternal and child primary health care programmes
4. To conduct an economic evaluation of the TBST strategy in comparison with the status quo, including its impact on health-related quality of life and on the capacity to perform economic activities among pregnant and lactating women

### Trial design and location

**Fig. 1:**
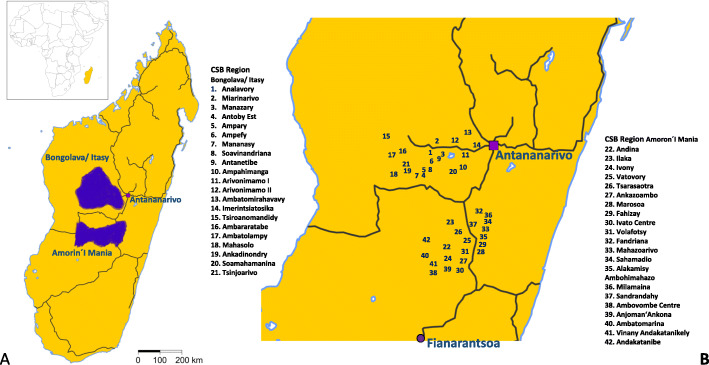
Distribution of the FreeBILy study sites across the regions of Bongolava, Itasy and Amoron’i Mania in Madagascar. In **A**, the geographic locations of these regions of Madagascar are depicted. In **B**, the full list of study sites and their distribution within the regions of Bongolava, Itasy and Amoron’i Mania are given

freeBILy in Madagascar is a two-armed 40-cluster randomized phase III trial assessing the potential impact of TBST on child growth and maternal haemoglobin (Hb). The 40 study centres are located in areas of medium to high endemicity of *S. mansoni* in Madagascar with 20 sites in the Itasy and Bongolava regions west of Antananarivo and 20 sites in the Amoron’i Mania region north of Fianarantsoa. The coordination institutions for these centres are the universities of Antananarivo and of Fianarantsoa (Fig. 1).

**Fig. 2:**
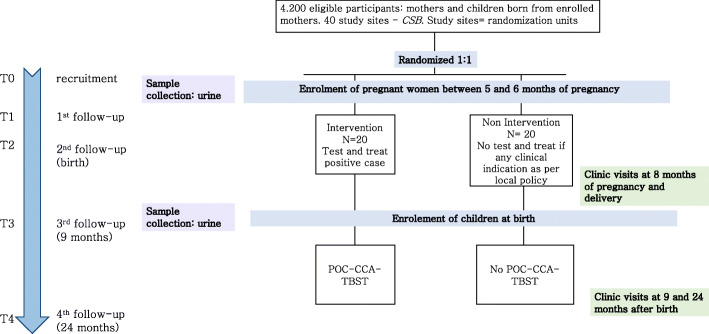
Cluster randomized trial study design. The CSBs, randomly allocated between intervention and non-intervention, represent the randomization units. At the 5th or 6th month of pregnancy (T0), women will be enrolled in the study and consent for their children will be asked. At T0 and 9 (T3) and 24 (T4) months after birth, urine samples will be collected. At delivery (T2), children will formally become part of the study

**Fig. 3:**
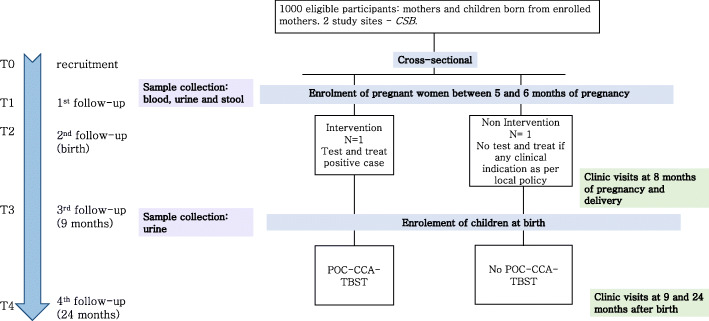
Design of the cross-sectional study. The two non-randomized CSBs Andina and Imerintsiatosika were selected as the intervention and non-intervention sites, respectively. At the 5th or 6th month of pregnancy (T0), women will be enrolled in the study and consent for their children will be asked. At T0, blood, urine and stool samples will be collected. At the end of the study, the sample will be analysed with the tests described in Table 3. At 8 months of pregnancy (T1) and 9 (T3) and 24 (T4) months after birth, urine samples will be collected. At delivery (T2), children will formally become part of the study

Figures 2 and 3 summarize the study design. According to the primary objectives, two different units of analysis exist: (i) children for whom the main outcome is the proportion showing stunted growth at 2 years of age and (ii) mothers for whom the main outcome is Hb level 2 years after delivery.

The units of randomization are the PHCC, called *Centres de Santé de Base* (CSB). There is a total of 23 CSBs in the Antananarivo and 49 CSBs in the Fianarantsoa study area. On the basis of the criteria listed in Table 1, 20 CSBs from the Antananarivo region (10 intervention and 10 control arms) and 20 CSBs from the Fianarantsoa region (10 intervention and 10 control arms) were selected. In addition to these 40 randomized CSBs, two non-randomized CSBs have been included in the trial: one is assigned to the intervention arm (Andina) and the other to the control arm (Imerintsiatosika). Objectives 1 and 2 are rolled out in the 40 randomized CSBs. Objectives 3 and 4 are rolled out in the two non-randomized CSB.

**Table 1:**
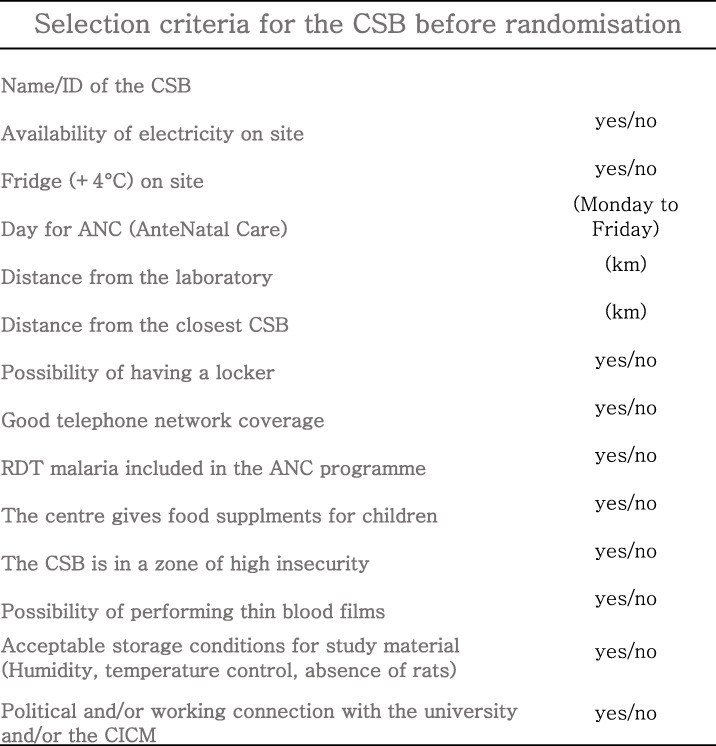
Inclusion criteria for the selection of CSB (Centre de Santé de Base). For all the CSBs, the mentioned criteria were assessed and on the basis of the best scoring the final 42 centres were selected

### Study setting

freeBILy in Madagascar is being implemented into routine maternal and child primary health care programmes, which consist of ante- and post-natal care, child routine examination and vaccination. In Madagascar, these programmes are established at CSBs, which are at the lowest administrative level of the health care system providing high accessibility to the population.

### Intervention

In study centres of the intervention arm, the POC-CCA-TBST is being implemented among pregnant women between week 20 of pregnancy and delivery and at 9 months after delivery and among their babies at 9 months of age. The urine-based POC-CCA-test is done, and PZQ treatment is offered in case of a positive diagnosis. In the study centres of the control arm, no TBST is carried out, and in case of clinical suspicion of schistosome infection, the participants are referred to the local health system. At the 24 months’ time point, TBST is being applied in both arms.

### Randomization

Randomization was done at the CSB level. For randomization, a list with the codes of the 20 CSBs in the regions of Bongolava and Itasy (so-called Antananarivo sites) and 20 CSBs in the region of Amoron’i Mania (so-called Fianarantsoa sites) was provided to the Leiden University Medical Center (LUMC), which randomly allocated 10 of the sites to control and 10 to intervention arm stratified by regions. LUMC informed the University of Antananarivo, University of Fianarantsoa and BNITM about the randomization result.

While blinding at the CSB level is not possible, all procedures of the study and of TBST (including urine sampling) are implemented at both the intervention and control CSBs. Only the actual POC-CCA test and the treatment of positives are exclusively done in intervention sites. In the participant ID, an alphanumeric element will define the recruitment site of the women. In case women would change the study site during the course of the study, the study nurses would be able to identify the non-correspondence of the site from the participant ID. This ensures that the randomization scheme is kept.

### Eligibility criteria

Eligibility criteria for the inclusion and/or exclusion of candidate participants are listed in Table 2. After information sessions at the research sites, eligibility criteria are preliminarily assessed for interested candidate participants. After the assessment, informed consent is requested to proceed with formal recruitment, which will start exclusively when informed consent is properly understood and signed by the participant. Informed consent will be obtained, from the participant or legal guardians of under 16 years old volunteers, by the study nurses who will enrol formally the participants just after signature.

**Table 2:**
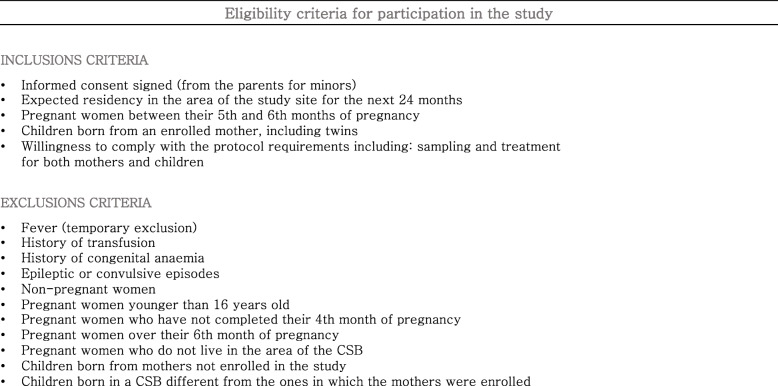
Eligibility criteria for the selection of the participants (pregnant women and later on children). The eligibility criteria are preliminary assessed after information sessions at the research sites. After assessment informed consent is requested to proceed with formal recruitment upon its reception

### Outcomes

The primary endpoints of the study are as follows:

1. The proportion of children showing stunted growth (height-for-age *z*-value < − 2) at 2 years of age to measure the impact of TBST on child development
2. Maternal Hb at 24 months after delivery to measure the impact of TBST on maternal health

The secondary endpoints of the study are as follows:

1. Sensitivity and specificity of the POC-CCA test compared to a panel of reference standard tests (measured as part of the cross-sectional study at non-randomized sites)
2. Women’s health-related quality of life, capacity to work and participate in economic activities and cost-effectiveness of the interventions

### Data collection plan

The baseline visit was conducted following informed consent and eligibility assessment. At enrolment, a unique identifier of 12 digits was assigned to each participant. The identifier is connected within the mother-child tandem and will be kept identical throughout the course of the study. Demographics and short medical history were obtained**.**

All sites have the same visit schedule. The sampling scheme is the same for the intervention and control sites; thus, the only difference between intervention and control sites is the TBST strategy at the intervention sites.

A POC-CCA-TBST is scheduled in the intervention arm at the following:

- Recruitment (5th to 6th month of pregnancy) and 9 months after delivery for the pregnant women/mothers
- Nine months of age for the children

A POC-CCA-TBST is scheduled in both arms at the following:

- Twenty-four months of age of children for both mother and child

The visit schedule of freeBILy is integrated into the mother and child care scheme offered to all pregnant women at the CSBs in Madagascar. freeBILy includes 5 different visits (T0, T1, T2, T3, T4) according to the following scheme as summarized in Table 3.

**Table 3:**
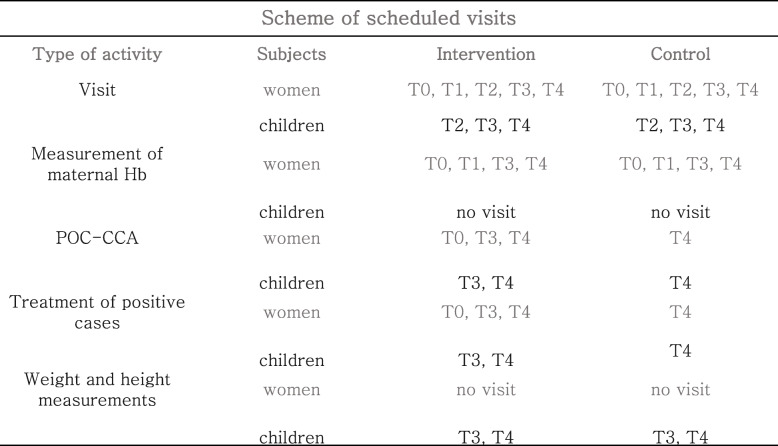
Scheme of scheduled visits. T0 mothers enrolment at 5^th^ or 6^th^ month of pregnancy pregnancy; T1 pre-delivery follow up at the 8^th^ month of pregnancy; T2 delivery follow up at birth and inclusion of children; T3 follow up 9 month after delivery; and, T4 final follow up 24 months after delivery

- T0: mother’s enrolment during the 5th or 6th month of pregnancy. Haemoglobin is measured on-site using the Hemocue® system, and a urine sample is being collected (≥ 25 ml). In the TBST intervention arm, the POC-CCA to test for schistosomiasis infection is done on-site. Women who test positive are offered (40 mg/kg) PZQ treatment. Additional samples (Table 4) are being collected at the non-randomized sites for the cross-sectional sub-study to assess the sensitivity and specificity of the POC-CCA test against a panel of reference tests described in Table 4.
- T1: 1st follow-up, 8th month of pregnancy. Haemoglobin is measured on-site by means of the Hemocue® system.
- T2: 2nd follow-up, delivery and child enrolment. The case report form (CRF) is completed. The newborns are included, unless the mother decides to withdraw her/him from the study. Weight and height of the newborns are measured.
- T3: 3rd follow-up, 9 months after delivery/birth. Haemoglobin is measured for all women using the Hemocue® system. Height and weight are measured for all children. A urine sample is being collected (≥ 25 ml) from all mothers and children. In the TBST intervention arm, the POC-CCA to test for schistosomiasis infection is being performed on-site for the mother and child. To test-positive mothers, a 40 mg/kg dose of PZQ treatment is offered. Test-positive children are treated with 300 mg.
- T4: 4th follow-up, 24 months after delivery/birth. Haemoglobin is measured for all mothers on-site using the Hemocue® system. Height and weight are measured for all children. A urine sample is being collected (≥ 25 ml) from all the women and children. In both arms, the POC-CCA test for schistosomiasis infection is being performed on-site for the mothers and children. All positive women and children are offered 40 mg/kg and 300 mg PZQ treatment, respectively.

**Table 4:**
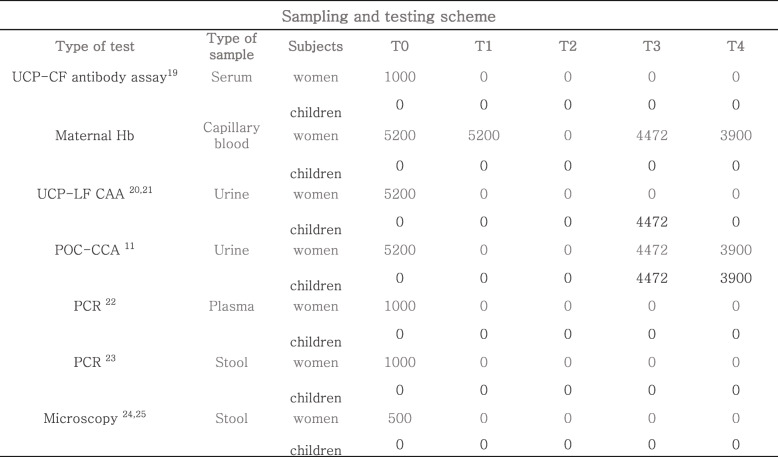
Scheme of scheduled visits. At T0 blood and stool samples will be collected from 1000 women, urine samples from 5200 women. At T1 capillary blood will be collected from 5200 women. At T2 no biological samples will be collected. At T3 and T4 capillary blood will be collected from all the women attending the follow-up visit, urine will be collected from all the participants attending the follow-up visit. Considering the loss to follow up 4472 mothers and 4472 children are expected at T3 to remain in the study and 3900 women and 3900 children at 4. UCP-CF (Up-Converting Phosphor – Consecutive Flow) antibody assay will be performed on serum samples. Hb will be measured on capillary blood. UCP-LF-CAA (Up-Converting Phosphor – Lateral Flow) and POC-CCA will be performed on urine samples. PCR will be performed on urine, plasma and stool samples. Microscopy will be performed on stool samples

All the samples collected for further laboratory analysis at the two non-randomized centres are stored on-site at the required temperatures and delivered to the laboratories within 1 week. Tables 4 and 5 summarize the testing and the sampling schemes. The tests will be performed according to standardized protocols described in [11–15, 18–25].

**Table 5:**
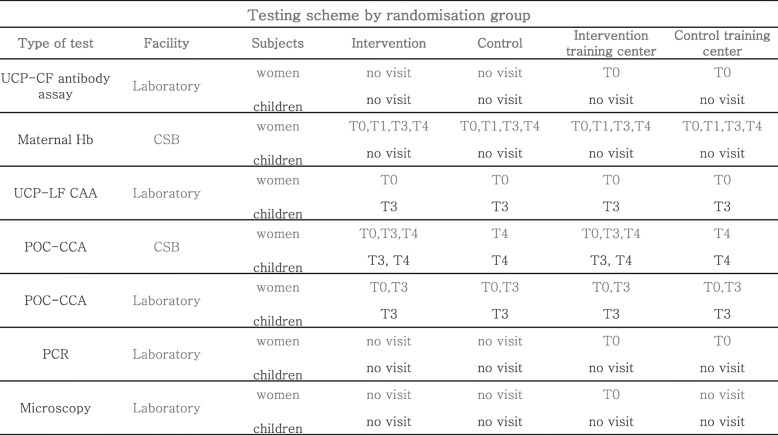
Testing scheme by randomization group. The UCP-LF antibody assay will be performed at the designated laboratory on all the samples collected at T0 from the women attending the visit at both intervention and control training centres. The maternal Hb will be measured at the CSB from all the women in all the sites attending T0, T1, T3 and T4 visits. The UCP-CF CAA and the UCP-LF CAA assays will be performed at the designated laboratory on all the samples collected at T0 from all the women in all the sites and at T3 from all the children in all the sites. The POC-CCA test will be performed for all the women attending the T0, T3 and T4 visits and for all the children attending T3 and T4 visits at the CSB belonging to the intervention group. The POC-CCA test will be also performed at the designated laboratory on all the samples collected at T0 from all the women in all the sites and at T3 from all the women and children in all the sites. PCR will be performed at the designated laboratory on all the samples collected at T0 from the women attending the visit at both intervention and control training centres. Microscopy will be performed at the designated laboratory on all the samples collected at T0 from the women attending the visit at the intervention training centre (one of the two non-randomised centres)

Information on women’s health-related quality of life is collected at T0, T1, T3 and T4 in a sub-sample of women enrolled at the non-randomized centres. Information on health system resources used for TBST is collected in all the facilities by means of a simple questionnaire administered to nurses involved in the intervention.

### Sample size and statistical analysis

The sample size was calculated in relation to the primary child outcome proportion showing stunted growth at 2 years of age, for which a larger sample size is assumed to be necessary than for the maternal outcome (Hb, 2 years after delivery).

The prevalence of stunting in Madagascar is estimated to be 40% of children under 2 years of age [26]. For our sample size estimate, it was conservatively assumed that:

- Twenty per cent of all children at the age of 2 years are stunted (height-for-age *z*-score < − 2)
- The intervention can reduce the proportion of stunted children by 50% in infected individuals, i.e. to observe that 10% of all children at the age of 2 years are stunted in the intervention arm.

In order to estimate the appropriate number of infected individuals at 9 months of age, sample sizes with power values of 80% and 90% were subsequently calculated using the OpenEpi tool [27]. For a power of 80%, at least 394 infected individuals at 9 months of age would be required and for a power of 90% 526 individuals. The design effect was assumed to be 1.9 by which the initial sample size needs to be multiplied to account for the clustered design of the study. This leads to a sample size of 749 for 80% power and 1000 for 90% power.

With an annual loss to follow-up of 10% from a visit to visit and an infant mortality rate of 4%, 86% of all mother-infant tandems are expected to present for the 9-month visit. For the 2 years visit, a loss to follow-up of around 10% between 9 months and 2 years is assumed, so that around 0.86 × 0.9 = 77.4% of all mother-infant tandems are expected to present for the 2-year visit. To correct for loss to follow-up, we use the factor 1/0.774 = 1.3. Since our analyses only focus on women infected with schistosomiasis, the number of women to be recruited needs adjusting, considering that the expected prevalence of 0.25 results in an additional inflation factor of 4 leading to an inflation factor of 1.3 × 4 = 5.2.

We hence planned to enrol 4200 eligible pregnant women in the 40 randomized cluster of the trial ensuring a power between 80 and 90% and an additional 1000 participants for the cross-sectional sub-study in the non-randomized sites. Based on the 40 randomized clusters identified for this study, an average of 105 women per cluster is needed to be recruited per cluster.

### Analysis for the primary outcomes

For the main child endpoint, the proportion of stunted children (height-for-age *z*-value < − 2) at 2 years will be compared between the intervention and control arm using the chi-square tests and *t*-tests. Reference standards from Madagascar as reported by the WHO [28] will be considered in the analysis. For the maternal main endpoint, the difference in Hb 2 years after delivery will be compared between the intervention and control arms using the *t*-tests. For all analyses, a significance level of alpha *=* 0.05 is assumed. Additional analyses will be performed using mixed-effects logistic and linear regression models to account for the between-cluster variation as a random effect. Secondary and other outcomes that are normally distributed will be analysed in the same way.

### Analysis for the secondary outcomes

To measure the sensitivity and specificity of the POC-CCA, the test results of the 1000 urines collected at the non-randomized sites at T0 will be compared to the results obtained from the reference panel (Table 5). Sensitivity of the POC-CCA is defined as the number of samples positive in the POC-CCA test divided by the total number of “true” positives (according to the results obtained from the combined panel of reference tests described in Table 5). Specificity of POC-CCA will be the number of sample negatives in the POC-CCA test divided by the total number of “true” negatives.

Additionally, women’s health-related quality of life scores will be compared between women in the intervention and in the control arms (Wilcoxon rank-sum test will be applied). A multivariate two-way fixed effects, regression model will be used to evaluate the effect of the intervention (while controlling for other factors) on women’s health-related quality of life scores. Finally, the costs of the intervention and health indicators will be used for the cost-effectiveness analysis. A decision tree model will be estimated and where the natural history of the disease will be represented by a Markov model. The model will be estimated by following standard approaches (i.e., deterministic and probabilistic analyses will be conducted). The association between women’s capacity to work and the intervention will also be estimated using the two-way fixed effects regression analysis, while controlling for observable time-varying factors.

### Trial governance

freeBILy was established as an international consortium in 2018 to focus on diagnostic alternatives to inform public health strategies to improve the management of schistosomiasis in high-endemic countries [16]. The Madagascar freeBILy study is being conducted under the direction of a Trial Steering Committee (TSC), which provides overall supervision for the trial on behalf of the sponsor, BNITM. The day-to-day operations and management of the trial are coordinated by the Trial Management Team (TMT) on the basis of a weekly meeting.

Internal and external monitoring measures are in place in order to ensure the proper management of the trial. Internally, the study is being regularly monitored by designated quality management staff in the field. In addition, at the end of the implementation phase and of each study phase (T0 to T4), a sponsor representative visits the study centres for monitoring purposes. The Data Safety and Monitoring Board (DSMB) provides independent expert oversight for the trial. It receives a monthly update of the recruitment and follow-up data and regular reports of serious adverse events (SAE). All possible side effects are recorded in a standardized form available at any time at the CSB and routinely reported to the Malagasy pharmacovigilance agency that acts as a surveillance body for the use of PZQ in the country. All participants are requested to wait at the CSBs for an hour after treatment in order to provide close monitoring of potential side effects. To date, the drug PZQ is not yet registered for pregnant women and U5 children. However, in more than 30 years of post-market experience with PZQ, no reports of serious adverse events relating to human pregnancy (e.g. abortions, stillbirths or congenital anomalies) have been published [29, 30].

### Ethical considerations

freeBILy in Madagascar is being conducted in line with the ICH (International Conference on Harmonisation)-GCP (Good Clinical Practice) guidelines, and findings are reported according to the Standards for the Reporting of Diagnostic Accuracy Studies (STARD) guidelines [31]. The study has been granted ethical approval by the National Ethics Committee of Madagascar (ref. no 022-SANP/CERBM of 05/03/2018) and the Ethics Committee of the Hamburg State Medical Chamber in Germany (ref. no PV5966 of 18/03/2019). Study participation is voluntary and on the basis of written informed consent. For pregnant women under the age of 16 and for children, informed consent is being sought from her mother or another legally designated representative. In all cases, participants/legally designated representatives have the right to refuse participation in the trial and are entitled to withdraw their informed consent, freely, at any time, without giving reasons.

### Community engagement and social mobilization

Community engagement and social mobilization activities were developed to engage the local population to facilitate study enrolment and decrease the loss to follow-up. General information sessions are being held for all women attending antenatal care services during their pregnancy. Discussion with local leaders was held in order for the community to endorse the trial. An information brochure was produced and distributed among the leaders of all different health facilities surrounding the study centres. Social workers met women in the community in order to discuss the trial and remind women of the follow-up visits. In case of participation rejection or withdrawal from the study, a questionnaire is being administered on a voluntary basis: this tool is allowing the team to adapt the recruitment and follow-up strategy in order to increase the chances of retention and final success of the study.

### Trial status

The current protocol version is 1.3 approved on 13 December 2019. The recruitment of pregnant women started on 4 April 2019 and was finalized on 4 February 2020. The trial is currently in the phase of its third follow-up visit in which 9-months children and their mothers are tested and treated for schistosomiasis. The follow-up will continue until autumn of 2022, when the child of the last included mother-child pair will have reached the age of 2 years. The present study aims at measuring primary outcomes on two distinct populations: pregnant women and young children. In this view, the whole study population is included in the study when also children are actively part of it. For this reason, it was decided to submit the study protocol towards the end of the T2 visit, in order to guarantee the full inclusion of the study population.

## Discussion

The overall aims of this study are to assess the potential impact of a POC-CCA TBST strategy on maternal and infant health and demonstrate the effectiveness of integrating the strategy at the primary health care level. MDA of PZQ is one of the main control measures against human schistosomiasis, but the success of MDA programmes is often hampered by low compliance with treatment, limited drug availability and misclassification of areas selected for MDA. Altogether, these factors lead to only partial coverage of the population in need [32]. There are claims for including pregnant women, infants and children under the age of 5 in high-endemic areas in MDA; however, they are usually not treated without diagnosis [5]. Diagnostic tools identifying infections at the PHCC level could help to integrate these vulnerable groups into control programmes.

Schistosomiasis is being listed as one of the neglected tropical diseases (NTDs), and the Sustainable Developmental Goals (SDGs) aim at having an elimination strategy for this NTD by 2030 [32, 33]. As we move towards elimination, and high prevalence areas may become low prevalence areas, it may be appropriate to move away from an MDA to a test-and-treat strategy [34]. This study assesses different aspects of a POC-CCA TBST strategy. We will assess if a POC-CCA TBST strategy can improve maternal anaemia and child development measured in terms of growth at 2 years of age. The study will show if an immediate benefit of this strategy can be shown for mothers and their children, who are usually excluded from PZQ-based treatment. The cost-effectiveness of the intervention will be assessed to support the development of national control strategies and guidelines for the treatment and management of schistosomiasis. More generally, the study offers the opportunity to help build capacity in conducting clinical trials in a setting with a high prevalence of NTDs and limited experience in clinical research.

The recruitment of pregnant women ended on 4 February 2020. So far, pregnant women are predominantly showing interest in the study and in the possibility of receiving the treatment during pregnancy. Our findings will be discussed with the local authorities in order to promote the integration of such strategy in national guidelines if the results will prove a positive impact on the target populations. If results will prove the effectiveness of the interventions, they could be easily translated to other countries where the endemic context of schistsomiasis is comparable to the one of Madagascar. Overall, our findings will contribute to an improved evidence base for the elimination strategy of schistosomiasis.

## Data Availability

Data will be available for the entire freeBILy consortium and for the scientific community upon request and validation from the trial steering committee.
